# Efficacy and safety of 1% and 2% rebamipide clear solution in dry eye disease: a multicenter randomized trial

**DOI:** 10.1186/s12886-023-03004-1

**Published:** 2023-08-03

**Authors:** Youngsub Eom, So Hyang Chung, Tae-Young Chung, Jae Yong Kim, Chul Young Choi, Kyung Chul Yoon, Byung Yi Ko, Hong Kyun Kim, Mee Kum Kim, Hyung Keun Lee, Jong Suk Song, Joon Young Hyon, Kyoung Yul Seo, Jong Soo Lee, Hyo Myung Kim

**Affiliations:** 1 Department of Ophthalmology, Korea University College of Medicine, Seoul, Republic of Korea; 2 Department of Ophthalmology, Korea University Ansan Hospital, Ansan, Gyeonggi-do Republic of Korea; 3 Department of Ophthalmology, Seoul St. Mary’s Hospital, College of Medicine, The Catholic University of Korea, Seoul, Republic of Korea; 4 Department of Ophthalmology, Samsung Medical Center, Sungkyunkwan University School of Medicine, Seoul, Republic of Korea; 5 Department of Ophthalmology, Asan Medical Center, University of Ulsan College of Medicine, Seoul, Republic of Korea; 6 Department of Ophthalmology, Kangbuk Samsung Hospital, Sungkyunkwan University School of Medicine, Seoul, Republic of Korea; 7 Department of Ophthalmology, Chonnam National University Medical School, Gwangju, Republic of Korea; 8 Department of Ophthalmology, Konyang University College of Medicine, Daejeon, Republic of Korea; 9 Department of Ophthalmology, School of Medicine, Kyungpook National University, Daegu, Republic of Korea; 10 Department of Ophthalmology, Seoul National University College of Medicine, Seoul, Republic of Korea; 11 Department of Ophthalmology, Seoul National University Hospital, Seoul, Republic of Korea; 12 Department of Ophthalmology, Gangnam Severance Hospital, Yonsei University College of Medicine, Seoul, Republic of Korea; 13 Department of Ophthalmology, Korea University Guro Hospital, Seoul, Republic of Korea; 14 Department of Ophthalmology, Seoul National University Bundang Hospital, Seongnam, Republic of Korea; 15 Department of Ophthalmology, Severance Hospital, Yonsei University College of Medicine, Seoul, Republic of Korea; 16 Department of Ophthalmology, Pusan National University College of Medicine, Pusan, Republic of Korea; 17 Department of Ophthalmology, Korea University Anam Hospital, Seoul, Republic of Korea

**Keywords:** Dry Eye Disease, Quinolones, Cornea, Tears, Intraocular pressure

## Abstract

**Background:**

To evaluate the efficacy of 1% and 2% rebamipide clear solution in the treatment of dry eye disease (DED).

**Methods:**

Two hundred twenty patients with DED were randomly assigned to one of three groups: the 1% rebamipide, 2% rebamipide, or placebo (eye drops containing the same ingredients, except for the active components). Each eye drop was instilled four times daily for 12 weeks. Changes in tear film break-up time (TBUT), corneal and conjunctival staining score, Schirmer 1 test, and the Ocular Surface Disease Index (OSDI) from baseline to 12-week visit between the study groups were compared for efficacy assessment.

**Results:**

The mean age of study patients was 43.8±14.2 years. The 1% and 2% rebamipide groups showed greater improvement in TBUT (1.99±1.87 and 2.02±2.21 s) at 12 weeks from baseline than the placebo group (1.25±2.93 s). The 2% rebamipide group showed greater improvement in the corneal staining score (− 3.15±2.00) at 12 weeks from baseline than the placebo group (− 2.85±1.80). The 1% and 2% rebamipide groups showed improvement in Schirmer 1 test (1.27±3.86 and 1.50±4.14 mm) at 12 weeks of treatment, but not the placebo group (0.55±2.99 mm). Both the rebamipide groups and the placebo group showed significantly improved OSDI after treatment for 12 weeks; however, there was no significant difference among the three groups.

**Conclusions:**

1% and 2% rebamipide clear solutions are an effective therapeutic option for improving TBUT and tear volume, and stabilizing the corneal staining score in DED.

**Supplementary Information:**

The online version contains supplementary material available at 10.1186/s12886-023-03004-1.

## Background

Dry eye disease (DED) occurs as a result of insufficient tear production or excessive evaporation of tears, and the symptoms include dry eye surface, discomfort, visual impairment, and ocular pain [1, 2]. Moreover, DED is characterized by increased osmolality of the tear film and inflammation of the ocular surface, and the prevalence is estimated at 5–30% in individuals aged 50 years or older [2–4]. DED is a serious condition that increases healthcare costs and decreases quality of life and work productivity, which leads to a substantial cost burden as well as an increased use of healthcare resources [5].

Currently, various treatment options are available for patients with DED depending on the severity of symptoms. Tear replacement products or punctal plugs are used to restore the homeostasis of the ocular surface and tear film [6–8]. Moreover, pharmacotherapeutic options have been recently developed to promote tear production and tear replacement combined with diverse types of lubricants aims to improve discomfort of the ocular surface [9, 10].

A novel quinolinone derivative, rebamipide, promoted wound healing in an experimental rat model of gastric ulcer [11, 12] Rebamipide has diverse biological effects in raising gastric endogenous prostaglandin E_2_ and I_2_, stimulating the secretion of gastric epithelial mucin, scavenging oxygen free radicals, and inhibiting inflammatory responses [13–15] Moreover, rebamipide is used to treat stomatitis, pulmonary, renal and liver damage, and colitis, [16, 17] and, in an in vivo model, protected the cornea [18].

Possible effects of rebamipide on mucin secretion on the ocular surface have been studied [19–22]. Non-clinical studies have shown that rebamipide stimulated the secretion of corneal and conjunctival mucin and actions of goblet cells in rabbits [20, 23–25]. Presumably, rebamipide’s therapeutic actions originate from an ability *not only* to stimulate the secretion of corneal and conjunctival mucin-like substances *but also* to improve corneal and conjunctival injury in vivo [18]. Furthermore, clinical studies have shown that rebamipide is effective in improving both damages to the corneal and conjunctival epithelium *as well as* patient symptoms [23]. Kukje Pharma (Gyeonggi-do, Korea) developed a novel transparent rebamipide eye drop (KSR-001) for the treatment of patients with DED. Therefore, this study was conducted to assess the efficacy and safety of this novel rebamipide formulation in Korean patients with DED.

## Methods

### Study design

This multi-center, randomized, double-blind, placebo-controlled, parallel group-comparison, phase IIb/III clinical trial, comprising a 2-week wash-out period followed by a 12-week double-blind treatment period, was conducted in 15 medical institutions in Korea between February 18, 2020 and February 8, 2021.

The study was approved by the Internal Institutional Review Board (IRB) of respective sites involved in it (2019AS0275) and then conducted in compliance with the relevant ethics guidelines. All the study treatments and procedures described herein were performed in accordance with the 1964 Declaration of Helsinki and its later amendments or comparable ethical standards. All participants provided written informed consent. The current study is registered with ClinicalTrials.gov (registration number: NCT05017844, first posted date: 24/08/2021).

### Study population

Patients aged 19 years or older, presenting with symptoms (e.g., foreign body sensation, dryness, glare, pain and blurred vision) suggestive of dry eye syndrome for ≥ 6 months before screening, and meeting the following criteria in one of both eyes at screening or randomization: corneal fluorescein staining score ≥ 4 points according to the National Eye Institute/Industry (NEI) scale and tear volume measured with Schirmer 1 test without anesthesia is ≤ 10 mm/5min (if Schirmer 1 test is 0 mm/5 min, then the result of Schirmer test with nasal stimulation should be ≥ 3 mm/5 min), and patients with best-corrected visual acuity (BCVA) of 20/100 or greater in the both eyes at both screening and randomization visits were included. Exclusion criteria for the current study are summarized in Table 1.

**Table 1:** Exclusion criteria for the randomized controlled trial

1. Patients for whom rebamipide therapy is expected to induce the onset of gastrointestinal disturbances or gastritis during the study. 2. Patients with a history of systemic steroid or immunosuppressant use within 90 days of the screening visit. 3. Patients with a history of using a punctal plug or undergoing ophthalmological surgeries, including punctal occlusion surgery, within 90 days of the screening visit. 4. Patients with clinically notable ophthalmologic diseases, other than DES, that may affect the results of the current study such as:
- Those with a history of corneal transplantation. - Those with a history of conjunctival scars due to cicatricial keratoconjunctivitis, herpes simplex keratitis, pterygium, pinguecula, congenital lacrimal gland agenesis, keratoconus, neurotrophic keratitis, or Sjogren’s syndrome. - Those with end-stage lacrimal gland disease (Schirmer’s test with nasal stimulation < 3 mm/5 min). - Those with blepharospasm, entropion, ectropion, or eyelash abnormalities. - Patients who need appropriate treatments for active ocular infections (e.g., anterior uveitis, blepharitis, or keratoconjunctivitis). - Patients who are being treated for allergic ophthalmologic diseases.
5. Patients with a history of laser vision correction surgeries, such as laser-assisted in situ keratomileusis (LASIK) or laser-assisted subepithelial keratectomy (LASEK), within 12 months of teh screening visit. 6. Patients with intraocular pressure (IOP) > 21 mmHg or those who are currently receiving pharmacological treatments for glaucoma at the screening visit. 7. Patients with hypersensitivity reactions to rebamipide, an active constituent of the study treatment. 8. Patients who plan to wear contact lenses during the study. 9. Patients meeting one of the following criteria at screening:
- Those with serum creatinine level ≥2× the upper limit of normal (ULN) - Those with serum aspartate aminotransferase (AST) / alanine aminotransferase (ALT) levels ≥2×ULN.
10. Patients with a history of malignancy, other than those with no recurrence within 5 years postoperatively. 11. Patients receiving treatment, after a diagnosis of alcohol or drug abuse within 1 year of screening. 12. Fertile women who did not agree to use medically approved methods of contraception, such as intrauterine device, intrauterine system, tubal ligation or double barrier contraception (a compound use of male condom, female condom, cervical cap, diaphragm or contraceptive sponge), during the study. 13. Women who are pregnant or breastfeeding 14. Patients with a history of using other drugs or devices in clinical trials within 30 days of study participation. 15. Patients who are deemed ineligible for study participation according to the investigator’s judgment

### Study protocol

After submitting a written informed consent for study participation at Visit 1, the participants were assigned a screening number. If determined to be eligible for study participation based on inclusion/exclusion criteria in accordance with the study protocol at screening, participants were not allowed to use eye drops, including treatment agents for DED, during the 2-week pre-study washout period. Any participant who did not use other eye drops before randomization and met inclusion/exclusion criteria at Visit 2 was randomized to either of three treatment arms, such as the 1% rebamipide, 2% rebamipide, and placebo groups. Each of the eye drop formulations was instilled four times daily for 12 weeks. Packages of clinical trial drugs was established and the randomization scheme was generated using SAS Version 9.4 or higher (SAS Institute, Cary, NC, USA), and the list was delivered to the interactive web response system (IWRS) developer and personnel who were responsible for the packaging of the clinical trial drugs. The randomization number and the batch number of clinical trial drugs were confirmed by the IWRS.

All the randomized participants were allowed to receive study treatments for 12 weeks, and were instructed to visit each study center at 4, 8 and 12 weeks for assessment of efficacy, safety, and eye tolerability.

### Study treatments

The study treatments were manufactured by Samil Co. Ltd. (Seoul, Korea) as requested by the Kukje Pharma and include the Study Treatment 1 (KSR-001-02; 1% rebamipide [rebamipide 10 mg/mL]), Study Treatment 2 (KSR-001-03; 2% rebamipide [rebamipide 20 mg/mL]), and Study Treatment 3 (KSR-001-04; 0% rebamipide [rebamipide 0 mg/mL]), all of which are colorless, transparent eyedrops dispensed in a translucent plastic container and stored in a tight container at room temperature (15–30 °C). The Study Treatment 3 (KSR-001-04) that contains the same ingredients except rebamipide was used as the placebo in the placebo group.

### Efficacy assessments

#### Tear break-up time (TBUT)

To evaluate tear film stability, the TBUT was measured three times with fluorescein paper strips (Haag-Sterit, Bern, Switzerland) while using a stopwatch, and the mean value was recorded up to the second decimal place.

#### Corneal Fluorescein staining (0–15)

Corneal fluorescein staining grade with fluorescein sodium-impregnated paper strips (Haag-Sterit) was evaluated according to the NEI Scale that relies on a chart that divides the cornea into five sections and assigns a value from 0 (absent) to 3 (severe) to each section, based on the amount, size, and confluence of punctate keratitis, to obtain a maximum score of 15 points [26].

#### Conjunctival Lissamine Green Staining (0–18)

Conjunctival lissamine green staining grade with lissamine green–impregnated paper strips (Contacare Ophthalmics & Diagnostics, Padra, India) was evaluated according to the NEI Scale [grades 0 (absent) to 3 (severe)] for each of the six areas on each conjunctiva, for a maximum score of 18 points [26].

#### Schirmer 1 test

To measure tear volume, the Schirmer 1 test without topical anesthesia was performed. After placing a filter paper strip inside the inferior-temporal conjunctival sac for 5 min, the wetted length (in millimeters) is measured.

#### Ocular surface Disease Index (OSDI) questionnaire (0–100)

To assess dry eye symptoms, a 12-item OSDI questionnaire, which consists of 3 subscales (ocular symptoms, vision-related functions, and environmental triggers) during a 1 week recall period was completed [27].

### Safety assessments

Differences in the BCVA and intraocular pressure (IOP) at baseline and the 12-week visit between the rebamipide treatment arms and the placebo group was the safety outcome measure. IOP was measured twice using non-contact tonometry, and if the difference between the two values was 2 mmHg or less, the average value was recorded. If the difference between the two values exceeded 2 mmHg, and additional measurement was obtained and the average value of the three measurements was recorded.

### Eye tolerability assessments

To evaluate eye tolerability of study treatments, seven symptoms (stinging/burning, itching, blurred vision, sandiness/grittiness, dryness, light sensitivity, and pain or soreness score) were evaluated at a grade from 0 to 3 (0, no symptom; 1, mild; 2, moderate; and 3, severe) after the instillation of study treatments.

### Participant assessment and criteria

For efficacy assessment, both the full-analysis and the per-protocol analysis were performed. The full-analysis set (FAS) comprised participants with available efficacy outcome data who received study treatments at least once after randomization. The per-protocol set (PPS) comprised participants of the FAS who had completed the current study without serious violation of the study protocol.

Safety analysis was performed for safety assessment, from the safety set which comprised participants who received study treatments at least once after randomization.

### Statistical analysis

The sample size was estimated based on previously published studies (Appendix A) [28, 29]. All data are expressed as mean ± standard deviation (SD) or the number of the participants with percentage, where appropriate. Difference in baseline characteristics of the participants between the rebamipide treatment arms and the placebo group were compared using one-way analysis of variance (ANOVA) and the chi-square test. Intragroup differences in efficacy outcome measures between baseline and the 12-week visit were compared using the paired *t*-test and Wilcoxon signed rank test in each group. Changes in efficacy outcome measured scores from baseline to the 12-week visit between the rebamipide treatment arms and the placebo group were compared using Wilcoxon rank sum test. Comparison of efficacy outcome measures between the baseline visit and each follow-up visit in each group were compared using the repeated-measures ANOVA with Tukey’s post hoc test. Differences in safety outcome measures at baseline and the 12-week visit between the rebamipide treatment arms and the placebo group were compared using one-way ANOVA. Eye tolerability symptom scores of both eyes at the 4-, 8-, and 12-week visits between the rebamipide treatment arms and the placebo group were compared using repeated-measures ANOVA. All statistical analyses were performed using SPSS ver. 23 (IBM corp., Armonk, NY, USA). A *p*-value < 0.05 was considered statistically significant.

## Results

### Patient demography

**Fig. 1:**
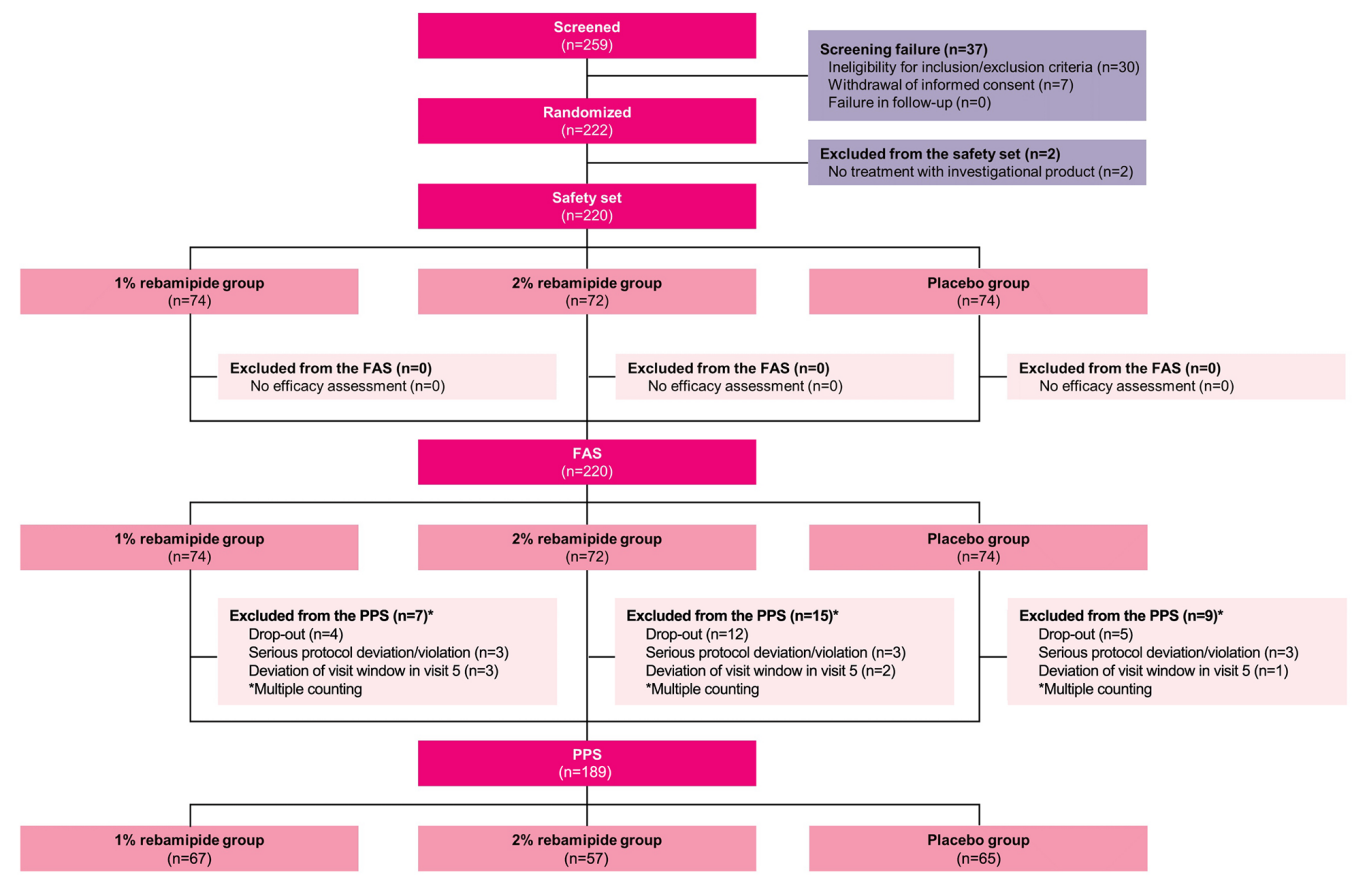
Flow diagram of a randomized controlled trial for assessing the efficacy and safety of 1% and 2% rebamipide clear solution in patients with dry eye disease

Among the 259 patients who were screened for this study, 222 participants were randomized to one of three treatment groups. Two patients were excluded due to non-administration of investigational drug and a total of 220 eyes of 220 participants (of 222 enrolled participants) – 74 in the 1% rebamipide group, 72 in the 2% rebamipide group, and 74 in the placebo group – were included in the FAS. In total, 189 (85.9%) participants completed the study as per protocol and were included in the PPS (Fig. 1). Of the 220 participants, 184 were female (83.6%) and the mean age ± SD was 43.8 ± 14.2 (range, 19–76) years. There were no significant intergroup differences in age, sex, height, and weight among the three study groups (Table 2).

**Table 2:** Baseline characteristics of the participants (n = 220)

Variables	1% rebamipide (n = 74)	2% rebamipide (n = 72)	Placebo (n = 74)	*p*-value*
Age, years, mean ± SD	44.2 ± 14.8	43.3 ± 13.6	44.0 ± 14.4	0.9297
Male:female, n (%)	10 (13.5):64 (86.5)	14 (19.4):58 (80.6)	12 (16.2):62 (83.8)	0.6251†
Height, cm	162.3 ± 6.5	162.3 ± 7.6	161.7 ± 7.4	0.8294
Weight, kg	58.6 ± 9.8	60.0 ± 10.4	60.5 ± 11.3	0.5084

* One-way analysis of variance

† Chi-square test

### Efficacy outcomes

**Fig. 2:**
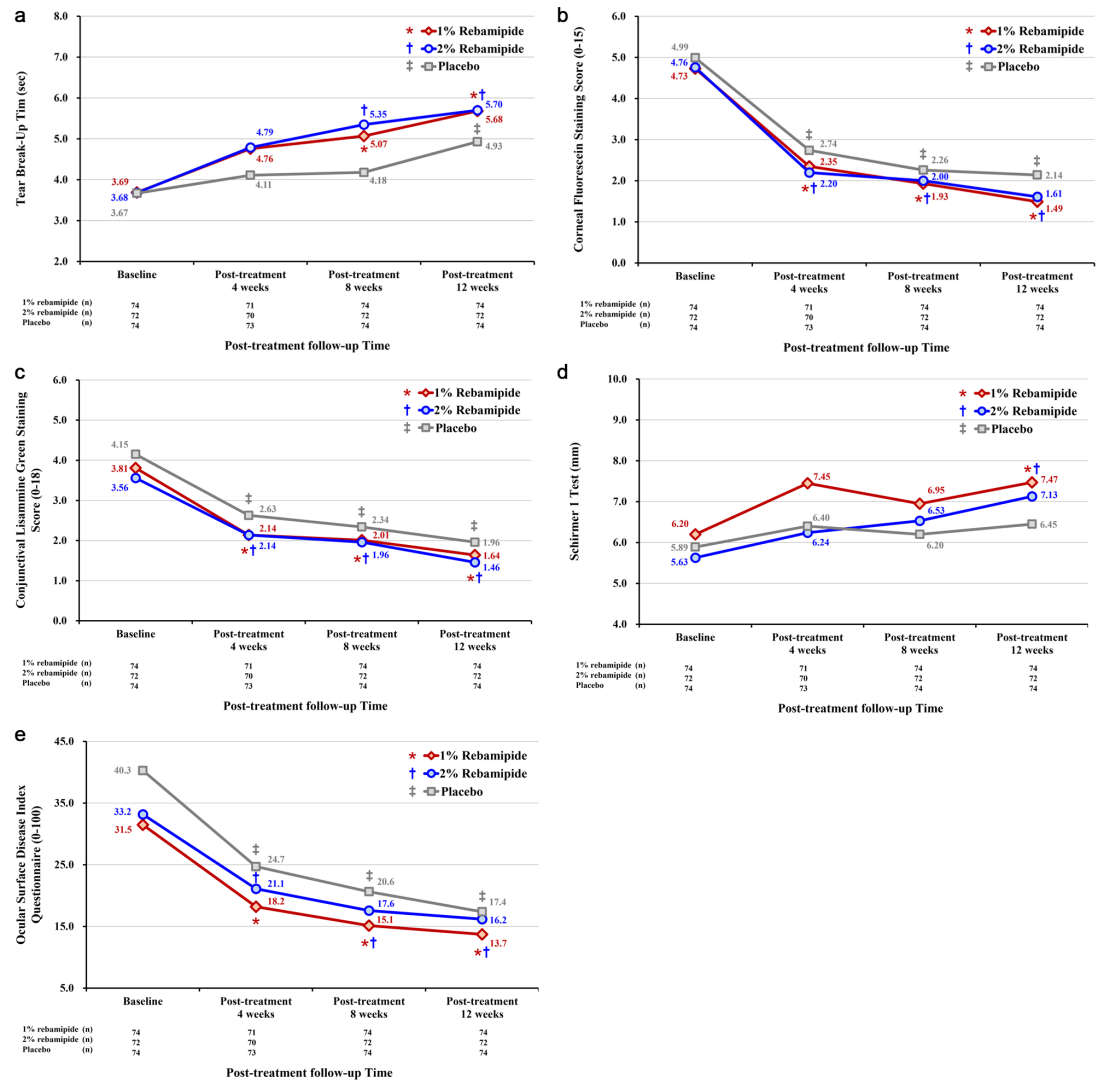
Comparison of tear break-up time **(A)**, corneal fluorescein staining score **(B)**, conjunctival lisammine green staining score **(C)**, Schirmer 1 test **(D)**, and Ocular Surface Disease Index questionnaire **(E)** at each visit among the three groups. The asterisks indicate significant changes in parameters from baseline to each follow-up visit in the 1% rebamipide group. The daggers indicate statistically significant changes in parameters from baseline to each follow-up visit in the 2% rebamipide group. The double daggers indicate statistically significant changes in parameters from baseline to each follow-up visit in the placebo group. Asterisks, daggers, and double daggers indicate *p* < 0.05 per repeated-measures ANOVA with Tukey’s post hoc test

The full analysis showed that, compared with the baseline, all the 1% and 2% rebamipide groups and the placebo group showed significantly improved TBUT, corneal fluorescein staining scores, conjunctival lissamine green staining scores, and OSDI after 12 weeks of treatment (all *p* < 0.0001). All three groups showed significantly improved corneal fluorescein staining scores, conjunctival lissamine green staining scores, and OSDI after 4 and 8 weeks of treatment, as compared with the baseline (all *p* < 0.05). However, at 8 weeks after treatment, the 1% and 2% rebamipide groups showed significantly improved TBUT compared with the baseline, whereas the placebo group did not (Fig. 2). Moreover, the 1% and 2% rebamipide groups showed significant differences in changes in TBUT at 12 weeks from baseline from the placebo group (*p* = 0.0148 and 0.0190, respectively). The 1% and 2% rebamipide groups showed improvement in Schirmer 1 test after 12 weeks of treatment, but not the placebo group. In addition, the 2% rebamipide group showed significant differences in changes in corneal fluorescein staining scores at 12 weeks from baseline from the placebo group (*p* = 0.0444). However, there were no significant differences in changes in conjunctival lissamine green staining scores and OSDI at 12 weeks from baseline between the rebamipide treatment arms and the placebo group (*p* = 0.6560, *p* = 0.4545, *p* = 0.1346, and *p* = 0.0908, respectively; Table 3).

**Table 3:** Comparison of efficacy outcomes between study groups in the full-analysis set (n = 220)

Variables	1% rebamipide (n = 74)	2% rebamipide (n = 72)	Placebo (n = 74)	*p*-value*	
**Tear break-up time, sec**					
Baseline	3.69 ± 1.78	3.68 ± 1.84	3.67 ± 2.96	1% rebamipide vs. Placebo	2% rebamipide vs. Placebo
12 weeks posttreatment	5.68 ± 1.86	5.70 ± 2.49	4.93 ± 2.77		
Changes in score	1.99 ± 1.87	2.02 ± 2.21	1.25 ± 2.93	**0.0148**	**0.0190**
*p*-value					
Baseline vs. 12 weeks visit	**< 0.0001**†	**< 0.0001**‡	**< 0.0001**‡		
**Corneal fluorescein staining score (0–15)**					
Baseline	4.73 ± 1.44	4.76 ± 1.32	4.99 ± 1.63	1% rebamipide vs. Placebo	2% rebamipide vs. Placebo
12 weeks posttreatment	1.49 ± 1.26	1.61 ± 1.67	2.14 ± 1.65		
Changes in score	−3.24 ± 1.80	−3.15 ± 2.00	−2.85 ± 1.80	0.0992	**0.0444**
*p*-value‡					
Baseline vs. 12 weeks visit	**< 0.0001**	**< 0.0001**	**< 0.0001**		
**Conjunctival lisammine green staining score (0–18)**					
Baseline	3.81 ± 3.03	3.56 ± 2.66	4.15 ± 3.34	1% rebamipide vs. Placebo	2% rebamipide vs. Placebo
12 weeks posttreatment	1.64 ± 1.75	1.46 ± 1.88	1.96 ± 2.22		
Changes in score	−1.68 ± 2.70	−1.49 ± 2.19	−1.58 ± 2.35	0.6560	0.4545
*p*-value‡					
Baseline vs. 12 weeks visit	**< 0.0001**	**< 0.0001**	**< 0.0001**		
**Schirmer 1 test, mm**					
Baseline	6.20 ± 2.49	5.63 ± 2.05	5.89 ± 2.33	1% rebamipide vs. Placebo	2% rebamipide vs. Placebo
12 weeks posttreatment	7.47 ± 3.78	7.13 ± 4.03	6.45 ± 2.93		
Changes in score	1.27 ± 3.86	1.50 ± 4.14	0.55 ± 2.99	0.4802	0.3180
*p*-value					
Baseline vs. 12 weeks visit	**0.0169**‡	**0.0026**‡	0.1152†		
**Ocular Surface Disease Index questionnaire (0-100)**					
Baseline	31.5 ± 15.9	33.2 ± 19.2	40.3 ± 18.3	1% rebamipide vs. Placebo	2% rebamipide vs. Placebo
12 weeks posttreatment	13.7± 15.3	16.2 ± 17.3	17.4 ± 16.0		
Changes in score	−17.8 ± 14.5	−17.0 ± 22.9	−22.9 ± 16.8	0.1346	0.0908
*p*-value					
Baseline vs. 12 weeks visit	**< 0.0001**†	**< 0.0001**†	**< 0.0001**‡		

* Wilcoxon rank sum test

† Paired *t*-test

‡ Wilcoxon signed rank test

The per protocol analysis showed that all participants in the 1% and 2% rebamipide groups and the placebo group showed significantly improved TBUT, corneal fluorescein staining scores, conjunctival lissamine green staining scores, and OSDI after 12 weeks of treatment (*p* < 0.0001). 12 weeks of treatment of 1% and 2% rebamipide significantly improved the Schirmer 1 test, but placebo did not. The 1% and 2% rebamipide treatment arms showed significant differences in changes in TBUT at 12 weeks from baseline from the placebo group (*p* = 0.0279 and 0.0055, respectively; Table 4).

**Table 4:** Comparison of efficacy outcomes between study groups in the per-protocol set (n = 189)

Variables	1% rebamipide (n = 67)	2% rebamipide (n = 57)	Placebo (n = 65)	*p*-value*	
**Tear break-up time, sec**					
Baseline	3.72 ± 1.81	3.51 ± 1.72	3.68 ± 3.11	1% rebamipide vs. Placebo	2% rebamipide vs. Placebo
12 weeks posttreatment	5.63 ± 1.87	5.71 ± 2.60	4.78 ± 2.44		
Changes in score	1.91 ± 1.85	2.20 ± 2.26	1.10 ± 2.83	**0.0279**	**0.0055**
*p*-value					
Baseline vs. 12 weeks visit	**< 0.0001**†	**< 0.0001**‡	**< 0.0001**‡		
**Corneal fluorescein staining score (0–15)**					
Baseline	4.76 ± 1.49	4.86 ± 1.42	4.94 ± 1.60	1% rebamipide vs. Placebo	2% rebamipide vs. Placebo
12 weeks posttreatment	1.55 ± 1.29	1.51 ± 1.53	1.92 ± 1.49		
Changes in score	−3.21 ± 1.85	−3.35 ± 1.91	−3.02 ± 1.66	0.3379	0.0567
*p*-value‡					
Baseline vs. 12 weeks visit	**< 0.0001**	**< 0.0001**	**< 0.0001**		
**Conjunctival lisammine green staining score (0–18)**					
Baseline	3.85 ± 3.16	3.63 ± 2.76	3.95 ± 3.32	1% rebamipide vs. Placebo	2% rebamipide vs. Placebo
12 weeks posttreatment	1.67 ± 1.79	1.37 ± 1.79	1.66 ± 2.03		
Changes in score	−2.18 ± 2.66	−2.26 ± 2.72	−2.29 ± 2.67	0.9908	0.3888
*p*-value‡					
Baseline vs. 12 weeks visit	**< 0.0001**	**< 0.0001**	**< 0.0001**		
**Schirmer 1 test, mm**					
Baseline	6.34 ± 2.45	5.42 ± 2.06	5.91 ± 2.36	1% rebamipide vs. Placebo	2% rebamipide vs. Placebo
12 weeks posttreatment	7.69 ± 3.91	7.07 ± 4.37	6.32 ± 2.75		
Changes in score	1.34 ± 3.95	1.65 ± 4.44	0.42 ± 2.93	0.3843	0.2658
*p*-value†					
Baseline vs. 12 weeks visit	**0.0212**‡	**0.0092**‡	0.2575†		
**Ocular Surface Disease Index questionnaire (0-100)**					
Baseline	31.1 ± 16.6	34.5 ± 19.4	41.0 ± 19.1	1% rebamipide vs. Placebo	2% rebamipide vs. Placebo
12 weeks posttreatment	13.4± 15.4	14.8 ± 15.3	18.0 ± 16.6		
Changes in score	−17.8 ± 14.5	−19.7 ± 22.1	−23.0 ± 17.0	0.1390	0.3295
*p*-value					
Baseline vs. 12 weeks visit	**< 0.0001**†	**< 0.0001**†	**< 0.0001**‡		

* Wilcoxon rank sum test

† Paired *t*-test

‡ Wilcoxon signed rank test

### Safety outcomes

There was no significant difference in the BCVA and IOP of both eyes at baseline and the 12-week visit between the rebamipide treatment arms and the placebo group (all *p* > 0.05; Table 5).

**Table 5:** Comparison of best-corrected visual acuity and intraocular pressure between the study groups (n = 220)

Variables	1% rebamipide (n = 74)	2% rebamipide (n = 72)	Placebo (n = 74)	*p*-value*
**Right-eye BCVA, logMAR**				
Baseline	0.04 ± 0.10	0.02 ± 0.07	0.03 ± 0.07	0.4134
12 weeks posttreatment	0.03 ± 0.12	0.02 ± 0.08	0.03 ± 0.07	0.7799
**Left-eye BCVA, logMAR**				
Baseline	0.02 ± 0.07	0.01 ± 0.05	0.03 ± 0.08	0.2414
12 weeks posttreatment	0.01 ± 0.07	0.02 ± 0.08	0.03 ± 0.07	0.2532
**Right-eye IOP, mmHg**				
Baseline	14.1 ± 3.0	13.9 ± 3.7	14.2 ± 3.2	0.8308
12 weeks posttreatment	14.3 ± 3.2	13.6 ± 3.3	14.1 ± 3.6	0.4260
**Left-eye IOP, mmHg**				
Baseline	14.3 ± 3.1	14.0 ± 3.7	14.7 ± 3.5	0.5259
12 weeks posttreatment	14.4 ± 3.0	13.7 ± 3.3	14.3 ± 3.4	0.3655

BCVA, best corrected visual acuity; logMAR, logarithm of the Minimum Angle of Resolution.; IOP, intraocular pressure.

* One-way analysis of variance

### Eye tolerability assessments

**Fig. 3:**
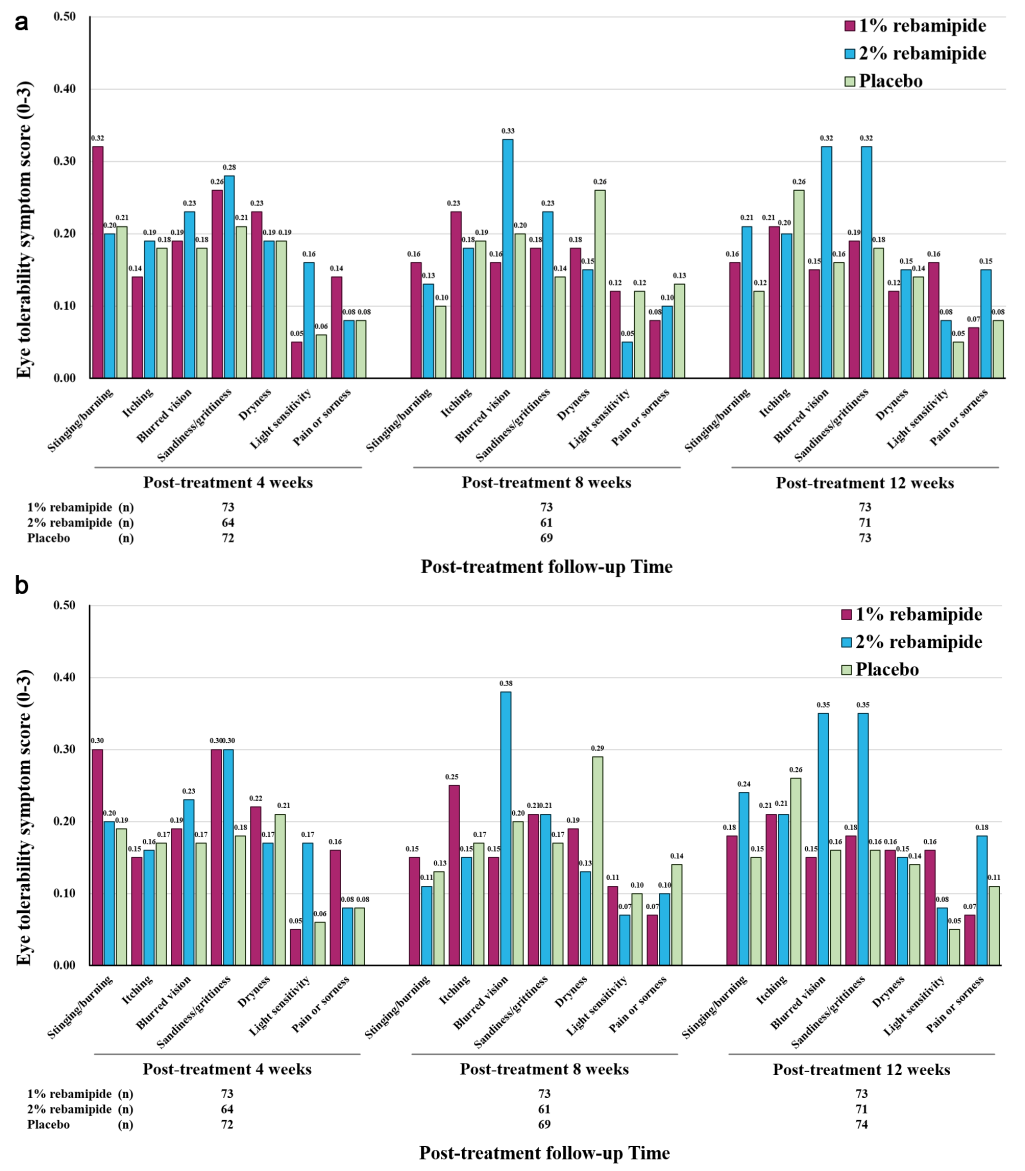
Comparison of the eye tolerability symptom score of the right eye **(A)** and the left eye **(B)** at each visit between the three groups. There were no significant intergroup differences in symptom score at each visit between the rebamipide treatment arms and the placebo group (all *P* > 0.05; repeated-measures ANOVA). One participant in the placebo group only reported symptom scores in their left eye at post-treatment 12 weeks

All symptom scores of stinging/burning, itching, blurred vision, sandiness/grittiness, dryness, light sensitivity, and pain or soreness after the instillation of study treatments of both eyes at the 4-, 8-, and 12-week visits of all three groups were less than 0.4. There were no significant differences in the seven symptom scores after the instillation of study treatments of both eyes at each visit among three groups (all *p* > 0.05; Fig. 3A and B).

## Discussion

This randomized controlled trial evaluated the efficacy and safety of 1% and 2% rebamipide clear solution in the treatment of Korean patients with DED. The results of this study showed that the 1% and 2% rebamipide clear solution resulted in greater TBUT improvement effect at 12 weeks of treatment compared to the placebo group. In addition, the 2% rebamipide clear solution showed significantly greater improvement in the corneal fluorescein staining scores at 12 weeks of treatment compared to the placebo group. The efficacy of rebamipide in improving TBUT and corneal fluorescein staining has been advocated previously in the literature [28]. Based on previous studies which showed that rebamipide was effective in stimulating the secretion of mucin-like substances on the cornea/conjunctiva, rebamipide is expected to stabilize the tear film and to improve the damage caused to the cornea/conjunctiva in patients with DED [18, 21].This is further supported by a large-scale, dose–response phase II study which showed that 1% and 2% rebamipide ophthalmic suspension significantly improved the TBUT and corneal fluorescein staining scores as compared with the control [28].

In this study, 12 weeks treatment of 1% and 2% rebamipide significantly improved tear volume represented as the Schirmer 1 test, but placebo did not. Unlike this study, previous randomized multicenter phase II and III studies evaluating the efficacy of 2% rebamipide solution haven’t show the improvement of Schirmer 1 test in dry eye patients [28, 29]. This difference is attributable to the difference in treatment period because rebamipide was used for 4 weeks in previous studies whereas rebamipide was used for 12 weeks in this study. There was no significant improvement in the Schirmer 1 test results at 4 and 8 weeks after treatment in this study. Previous study which evaluated the effect of rebamipide ophthalmic solution on dry eye disease mice showed that the treatment of rebamipide increased not only the conjunctival goblet cell density but also the tear volume represented as the phenol red test [30]. Therefore, according to the results of this study, if rebamipide is used continuously for more than 12 weeks, it is thought that DED can be improved by increasing tear volume as well as mucin layer of tear film.

According to the Korean Corneal Disease Study Group (KCDSG) guidelines for the diagnosis and treatment of DED, patients presenting with at least ocular or visual symptoms as well as one of three objective signs, such as corneal fluorescein staining scores, TBUT, and Schirmer test, can be diagnosed with DED. This study intended to enroll dry eye patients with dry eye Levels I, II, or, III, as defined by the KCDSG guidelines. Thus, patients who presented with symptoms suggestive of dry eye syndrome for ≥ 6 months and with increased ocular surface staining and decreased Schirmer 1 test were enrolled. However, TBUT was not included in the inclusion criteria as most dry eye patients had short TBUT in South Korea and Japan in previous studies [31–34]. Although there was no inclusion criterion related to TBUT, the mean TBUT of the three groups in this study were 3.69 ± 1.78, 3.68 ± 1.84, and 3.67 ± 2.96 s.

The treatment recommended for patients with DED includes inoculation of artificial tears or anti-inflammatory agents (e.g., steroid or cyclosporine eye drops) [35]. However, these treatments have limited efficacy because artificial tears may produce transient effects or the frequent use of eye drops containing preservatives may irritate the epithelial cells or cause epithelial damages [36]. Steroid eye drops are effective in treating inflammation of the eyelid and cellular injuries, but they may increase the risk of infections, increased IOP, and cataract [37]. Moreover, patients with DED commonly stop using treatment agents after they achieve a partial recovery from their symptoms [38]. It is therefore imperative that a novel treatment agent be developed to stimulate continuous secretion of mucin on the ocular surface and thereby stabilize the tear film and to improve the damage to the ocular surface.

Diquafosol tetrasodium and rebamipide are well-known mucin secretagogues, and both are approved for the treatment of DED in Japan [39, 40]. Diquafosol, a purinergic P2Y2 receptor agonist, not only enhances tear fluid production from conjunctival epithelial cells but also promotes mucin secretion from conjunctival goblet cells because purinergic P2Y2 receptors have been identified in conjunctival epithelial and goblet cells [40–42]. The main mechanisms of rebamipide are to stimulate prostaglandin and mucus glycoprotein synthesis while inhibiting inflammatory cytokines, reactive oxygen species, and neutrophil activation [15]. In addition, rebamipide promotes the growth of conjunctival goblet cells through the activation of the EGFR-signaling pathway, which is linked to goblet cells,[25] and increases MUC5 mRNA expression on the ocular surface [43] However, 2% diquafosol and 2% rebamipide did not meet the data requirements for regulatory approval and have not been approved by the FDA [39]. Thus, although this study showed that the newly developed transparent 2% rebamipide are effective in the treatment of DED, the 2% rebamipide formulation that has been used in this study may not satisfy the FDA’s requirements, and additional research on the effect in DED is needed.

Patients with DED are vulnerable to ocular discomfort and irritation, burning sensation, itching, and blurred vision [44, 45]. Moreover, these patients are at a risk of decreased functional visual acuity,[46] which may greatly affect social and physical functioning, workplace productivity, and quality of life in patients with DED [47, 48]. In the current study, rebamipide treatment arms showed no significant differences in BCVA and IOP at 12 weeks compared with the placebo group. In addition, there were no differences in the eye tolerability symptom scores after the instillation of study treatments at each visit among three groups. Taken together, these results attest to the safety of 1% and 2% rebamipide treatments in patients with DED.

In this study, both 1% and 2% rebamipide clear solutions and placebo significantly improved all signs and symptoms except Schirmer 1 test in the efficacy assessment after 12 weeks of treatment. When studying the effect of topical eye drops, a vehicle that resembles tear substitutes could improve signs and symptoms of patients. Nevertheless, in this study, rebamipide clear solution showed the effect of significantly improving TBUT, Schirmer 1 test, and corneal fluorescein staining compared to the placebo group.

## Conclusions

In conclusion, although there was no significant difference between the rebamipide treatment arms and the placebo groups in the improvement of OSDI after 12-week treatment, this study demonstrated that 1% and 2% rebamipide clear solutions are an effective, safe therapeutic option for improving TBUT and tear volume, and can stabilize the ocular surface in patients with DED.

### Electronic supplementary material

Below is the link to the electronic supplementary material.

Supplementary Material 1

## Data Availability

The datasets during and/or analysed during the current study available from the corresponding author on reasonable request.

## List of abbreviations

DED: dry eye disease
IRB: institutional review board
NEI: National Eye Institute/Industry
BCVA: best-corrected visual acuity
IWRS: interactive web response system
TBUT: tear film break-up time
OSDI: Ocular Surface Disease Index
IOP: intraocular pressure
FAS: full-analysis set
PPS: per-protocol set
SD: standard deviation
ANOVA: analysis of variance
