# Supplementary material for: Efficacy and safety of 1% and 2% rebamipide clear solution in dry eye disease: a multicenter randomized trial
Source: BMC Ophthalmol. 2023 Aug 3;23:343. doi: 10.1186/s12886-023-03004-1 (PMC10398964; doi:10.1186/s12886-023-03004-1)
Supplement: Supplementary file 1 — Supplementary Material 1 [file 12886_2023_3004_MOESM1_ESM.doc]

**Appendix A. Rationale of sample size estimation.**

Two previous studies have shown a mean difference of 1.7 from the baseline for changes in the corneal fluorescein staining scores between the 1% rebamipide treatment group and the placebo group at the endpoint.[1, 2] Moreover, the standard deviation (2.89) seen in the 2% rebamipide group served as a standard deviation in the current study.[2]

To verify whether there was a higher degree of changes in corneal fluorescein staining scores in the trial group than in the placebo group, a level of significance (α = 0.05), statistical power (1−β = 0.9), a trial group-to-placebo group ratio (λ = 1), the standard deviation in both the trial and the placebo groups (σ_t_ = σ_p_ = σ = 2.89), differences in mean changes in corneal fluorescein staining scores between the trial and the placebo groups (1.7), and a drop-out rate of 12% were used. The number of participants per group could be calculated based on the following formula:

n = (2σ^2^[Z_1-α/2_Z_1-β_]^2^)/(μ_t_-μ_p_)^2^

Therefore, the number of participants per group was estimated as 61. Considering a dropout rate of 12%, 70 participants were planned for assignment in each treatment arm.

**References**

1. Kinoshita S, Awamura S, Oshiden K, Nakamichi N, Suzuki H, Yokoi N: **Rebamipide (OPC-12759) in the treatment of dry eye: a randomized, double-masked, multicenter, placebo-controlled phase II study**. *Ophthalmology* 2012, **119**(12):2471-2478.

2. Kinoshita S, Oshiden K, Awamura S, Suzuki H, Nakamichi N, Yokoi N: **A randomized, multicenter phase 3 study comparing 2% rebamipide (OPC-12759) with 0.1% sodium hyaluronate in the treatment of dry eye**. *Ophthalmology* 2013, **120**(6):1158-1165.
